# On the Origin and Mode of Development of Tubercles

**Published:** 1839-07-13

**Authors:** W. W. Gerhard


### CLINICAL LECTURE.

### ON TUBERCULOUS DISEASE.

LECTURE I.—*On the Origin and Mode of Development of Tubercles.*

By W. W. GERHARD, M. D.

THE tuberculous disease is, to an eminent degree, variable in its symptoms, and is sometimes so much concealed by other adventitious disorders, that the original character of the affection is apparently lost, or, at least, is discovered with great difficulty. This arises from the fact that the symptoms of the general tuberculous disease are extremely obscure, while the local signs indicative of the affection of the organs in which tubercle is developed are often very strongly marked; hence we very easily forget that the disease itself is a unit, and that the local symptoms are, for the most part, purely secondary, and arise from several different causes. These local signs are, in part, such as are produced by the deposit of any adventitious matter, which necessarily excites a degree of irritation in the organ, in the effort to throw it off, proportionate to the quantity secreted; in part they arise from active determination of blood, and excitement of the organ, which occurs during the secretion of the adventitious substance.

The artificial divisions of the tuberculous disease, which are derived from the organs in which this substance is deposited, are so numerous, that you will have some difficulty in discovering the connecting link. The most frequent and one of the most fatal of these disorders is phthisis pulmonalis, or consumption of the lungs; in this disease, as is well known, the tubercles are seated in the pulmonary organs, and excite so much cough and other local disturbance, as to give the affection the common appellation, disease of the lungs, as synonymous with tuberculous disease of those organs. This is the most frequent of the severe chronic diseases of these organs. Next in frequency is acute hydrocephalus of children, from the age of one or two years to puberty; it sometimes occurs in adults, but less frequently. In the latter case, it is almost always connected with a tuberculous disease of the lungs, while in children this connection is not so frequent. There is another common tuberculous disease, which has received various appellations. It is those cases in which tuberculous matter is secreted in the peritoneum and in the mesenteric glands of the abdomen, so as to give rise to chronic inflammation of its serous membrane. Frequently you will find this disease designated as tabes mesenterica, sometimes as chronic peritonitis, &c. The various forms of scrofulous disease of the lymphatic glands are also characterized by the deposit of tuberculous matter in their early or later stages. Besides these recognised diseases, nearly all the organs of the body are more or less exposed to the tuberculous deposition; but as their symptoms are less connected with the disorder of important functions, these lesions are either passed over without notice, or they are regarded as merely accidental deposits, which occur during the course of those varieties of tuberculous disease which have received distinct names.

All these varieties of the tuberculous disease offer a singular uniformity in their anatomical characters. In all, you will find that the essential character is the secretion of tubercle. We apply this term to rounded yellow bodies, of nearly the hardness of cartilage, perfectly homogeneous, offering no trace of bloodvessels, and varying from the size of a mustard seed to that of an almond; they are most evident when they have attained the size of peas. You will get the clearest idea of the character of tuberculous matter from these tubercles of a medium size; but the substance is, of course, found in several different degrees of development. In its commencing stage there are two distinct forms; one is that in which the tubercle is obviously at the very commencement rounded, and, as it were, isolated. It assumes the form of a small round body at first, hardly so large as the head of a pin. In the lungs you can scarcely detect the isolated tubercle, unless it has attained this size; but in the serous membrane, we have from the great transparency of this tissue much better opportunities of recognising its forming stage. In these membranes we detect the tubercle as a small point, just visible to the unassisted eye; with the microscope you will find that this point is in its substance perfectly free from vessels, and that it is composed of a homogeneous substance without globules,—in other words, that it is closely analogous to albumen. The surrounding serous membrane is in general free from tubercles, unless they are visible to the naked eye; hence we may conclude that the action of the vessels necessary to the secretion of tubercle, gives rise to a tuberculous granulation of a determinate size, and very rarely to a minute microscopic deposit. The tubercle is deposited in the inner, that is, the adherent layers of the serous coat,—the delicate, smooth membrane, which may be considered as the proper serous coat, evidently passes over the tubercle.

The colour of these bodies is always an opaque whitish tint, never at the commencement yellow. But in advanced cases, we very often detect in one portion of the serous coat a number of tubercles, which are already yellow and opaque, and others still white and semi-transparent. There is, therefore, no doubt that the opinion of those who regard the grayish or white granulations as the early stage of tubercle, is correct. The form of the granulations in the serous membranes is rounded, but a little flattened and spread out upon the membrane. They very rarely pass to the period of softening, but in general, if they increase to a large size, adhesions take place between the two surfaces of the pleura, and no effusion of the softened matter takes place into its cavity.

The serous membranes afford us the best opportunity of studying the form of tubercles at their earlier period, and from the same transparency enable us to decide, with tolerable precision, the vexed question of the inflammatory or non-inflammatory origin of tubercles. In my demonstrations I have very frequently pointed out these cases of tuberculous inflammations of the serous membranes, and you have traced with me the different steps of their progress. When the case is acute, it is much more obviously inflammatory than in those which are slower. These acute cases are attended with considerable febrile excitement, which sometimes becomes violent, and not a few patients perish during the early period of the disease, from the immediate effects of the pleurisy, and not from phthisis. Upon examining the pleura in these cases, you will find that the semi-transparent granulations are surrounded by a red circle formed by the blood-vessels developed during the course of the pleurisy. The vessels are most numerous in those parts of the pleura where the tubercles are largest, and placed nearly in contact. The vessels are arterial, and are visible to the naked eye; there are, however, others much smaller, and only distinguishable by a microscope. These vessels evidently do not enter the tubercle, but merely surround it, and perhaps communicate with the investing capsule. The smallest granulations, like the largest, are surrounded by the same circle of vessels, and are evidently secreted in the same manner. The pleura may be detached from a portion of the granulations, but others come off with it. This arises from the fact that the lamina constituting the pleura proper does not enter into their substance; it is only the intermediate, and, as it were, cellular coats of the pleura, which nourish the newly-formed structure.

Besides the pleura proper, the false membrane, or coating of lymph, effused upon and within the serous membrane, is often the seat of tuberculous deposit. This never takes place except when the false membrane contains numerous red vessels, and is evidently in a state of active inflammation. It scarcely happens that tubercles are found in false membranes, except in connection with these evident marks of inflammation; where they are met with under other circumstances, the membranes have contracted firm adhesions with the pleura, and have lost their distinctive character of newly-formed lymph. The development of tubercle in false membranes is rarely observed, except in the most acute cases of the disease; indeed, the same remark applies to tubercles of serous membranes in general, which rarely present these bodies except in some acute form of the tuberculous disease.

The evidence of inflammation, then, is conclusive in these cases: we have the bright vivid, red injection, as well as the peculiar products of inflammation, such as lymph and pus, or at least puriform liquid. The tubercles are evidently secreted from vessels in a state of active turgescence, and that, too, in membranes which in their natural state are altogether deprived of vessels carrying red blood. We must conclude, then, that tubercle in certain cases is the result of an inflammatory action of a very intense character.

I have selected the case of tubercles of serous membranes merely on account of the clear demonstration of the steps of the process which is furnished by a transparent membrane; but in the other tissues of the body the same rule holds good. In tuberculous disease of the follicles of the intestinal canal, particularly of the glands of Peyer, I have traced the same vascular development at the commencement of the disorder. The injection was nearly as intense as in the serous membranes; at least, the difference was not greater than is usually observed in the inflammations of these tissues. The inflammation was not secondary, because it was developed at the very commencement of the lesion, when the most minute points of tuberculous matter could be detected, and was by no means confined to the cases in which the granulations had attained a considerable size. In the lungs, in certain cases, the same inflammatory process is quite evident; but, in these organs it is chiefly confined to two varieties. In one of these varieties, you find a large portion of the tissue of a bright red tint, rather more vermillion than in ordinary cases of pneumonia, and thickly interspersed with semi-transparent granulations of a remarkably equal size, as if they had been secreted simultaneously; or, as it were, by the action of the vessels. In another variety, the lung is studded with nodules of a darker red, composed of one or more lobules and quite hard; these nodules are found to contain an immense number of semi-opaque granulations; apparently deposited in the vesicles themselves, and contrasting, by their dull white or yellowish tint, with the redness of the proper pulmonary tissue. In the lymphatic glands, in the brain, especially its membranes, and occasionally in other organs of the body, the same coincidence of unequivocal inflammation is often met with, and nearly always the tuberculous disease is of the acute form, and terminates very quickly.

The question which next arises is, whether this deposit of tubercle is strictly identical with inflammation, or whether it offers characters of a specific kind. That it is a true inflammation is very clear, for it not only presents its usual anatomical characters, but the disease evidently arises from the same causes as commonly give rise to inflammation, and follows the same order of symptoms; but, it is equally evident that the majority of cases of inflammation of organs are not accompanied by a secretion of tubercle. Hence, there is certainly some other cause at work, the intimate nature of which is unknown. This cause, it would seem, is, at times, developed suddenly, and is accidental; at other times, it is inherent in the individual, born with him, and constitutes a part of his nature. It is then called the tuberculous diathesis, and constitutes the predisposition to this class of diseases, which descends from parents to their offspring.

You perceive, then, that in the evidently inflammatory and acute forms of tuberculous disease, we are compelled to admit an unknown something, different from ordinary inflammation, and, as it were, added to it. In the more chronic forms of the same affection, the influence of the inflammatory action becomes more obscure, and in not a few cases is altogether doubtful, perhaps improbable. It is, then, more strictly, or, at least, more evidently, a constitutional disease, developed throughout the whole system, and apparently connected with a generally deteriorated state of the nutrition. It does not materially differ from the more acute forms, except that the evidence of local inflammation is less, and that of the general diathesis is greater. Are we, then, at liberty to conclude that there is no inflammatory action whatever? This is the doubtful point in the history of tubercle, and cannot be decided, except we first clear it from the obscurity which results from a misunderstanding as to the precise meaning of the term.

If we limit the term inflammation to those cases in which there is vivid injection of the vessels around the tuberculous secretion, it is very certain that in the majority of cases of chronic forms of tubercle this state of the parts does not exist. It is true, that in the sub-acute forms there is not unfrequently some injection of the vessels around the tuberculous matter; but in the chronic varieties of the tuberculous disease, the surrounding tissue is often even paler than natural, and is sometimes quite endemic. These chronic forms are usually developed in patients who have been placed under circumstances tending to enfeeble the constitution and reduce the nutritious powers of the blood, and, therefore, the signs of inflammation must necessarily differ from those of the same morbid action when it occurs in healthy and tolerably robust individuals. You are aware that pus and lymph are often secreted in the serous membranes of very bloodless and exhausted patients, without a trace of redness of the vessels, and we cannot, therefore, lay much stress upon the absence of the usual redness of inflammation, except as a presumptive reason against the existence of this process.

We are obliged to examine the question under other points of view, and we find that the proofs of the existence of inflammation are not to be discovered in a great proportion of cases of chronic tuberculous disease. In these cases the disease takes place over a great number of different portions of the body, and is usually slow in its progress in most of the affected organs. The health of the patient is slowly deteriorating, while there is no adequate sign of local inflammation, or even in many cases of a decided change in the functions of any important organ. It is only late in the disease, when the local deposits of tubercles are so numerous as to interfere with the functions of an organ, and excite a secondary inflammation by acting as a foreign body, that unequivocal signs of inflammation appear; hence we have no right to conclude that in these cases tubercles are the result of an inflammatory action.

I have stated, when speaking of the acute form of tuberculous disease, that although the secretion was evidently connected in most cases with an inflammatory action, it was impossible to explain the progress of the disease upon these grounds only. We are obliged to admit the existence of a constitutional peculiarity, which adds to pus, the ordinary product of inflammation, the new formation, tubercle. In the chronic forms of the disease, we have seen that the local signs of inflammation either totally disappear, or are so much obscured as to make it evident that this process has little or no influence in the formation of tubercle. We are, then, obliged to admit, that the unknown constitutional peculiarity which is called into action in the acute cases by an inflammatory process, is sufficient in the chronic cases to develope tubercle, without the usual phenomena of inflammation.

We are utterly ignorant of the nature of this peculiarity, but we know the circumstances by which it is most usually preceded. These are such as depress the forces of the economy, and impoverish the blood. These causes are, of course, numerous, and you will find them enumerated in most works on medicine. Amongst them you will class attacks of fever, sedentary occupations, deficiency of air and light, a scanty diet, and depressing passions of the mind. The latter cause you should bear constantly in mind; for whenever a patient, disposed to phthisis, is suddenly deprived of his hopes of success and happiness in life, a tuberculous disease is a very usual consequence, and death often results. I was much struck with a case of the kind which I witnessed a few years since. A young man, an assistant engineer on our public works, met with an injury of the ankle-joint. It was very slight, and no appreciable change in the appearance of the joint could be detected, but he suffered severe pain when he attempted to walk. As a necessary consequence, his business was interrupted, and in the following year phthisis set regularly in.

I do not intend, in the present lecture, to enter more largely into the causes of tubercle. I was obliged to make some allusion to them, in order to impress upon your mind the two distinct ways by which the tuberculous diathesis is awakened into actual secretion. In the one, a local inflammation induces the secretion of tubercle, in addition to the usual products of inflammation. In the other this matter is deposited from the capillaries, in the substance of organs which seem to act as mere strainers, or to exercise a sort of disposing affinity upon the tuberculous matter, which is probably contained in the blood of patients subjected to the influences of which I have already spoken. The difference between the two modes of secretion of tubercle is nearly analogous to the two modes in which pus is deposited in the organs; in the more common mode there is a local phlegmasia, which terminates in abscess; in metastatic abscess of the lungs, on the other hand, the deposit of pus is made directly from the blood, and the proper inflammation is altogether secondary.

These opinions may seem to you hypothetical. They are not, perhaps, rendered as clear as they might be, nor is the chain of proof complete. In most respects, however, you may be assured of their correctness.

